# Excitatory neurotransmitters in brain regions in interictal migraine patients

**DOI:** 10.1186/1744-8069-5-34

**Published:** 2009-06-30

**Authors:** Andrew Prescot, Lino Becerra, Gautam Pendse, Shannon Tully, Eric Jensen, Richard Hargreaves, Perry Renshaw, Rami Burstein, David Borsook

**Affiliations:** 1 Brain Imaging Center, McLean Hospital, Belmont, MA 02478, USA; 2 P.A.I.N. Group, McLean Hospital, Belmont, MA, USA; 3 P.A.I.N. Group, Martinos Center, Massachusetts General Hospital, Charlestown, MA, USA; 4 Imaging, Merck & Co, West Point, PA, USA; 5 Department of Anesthesia, Beth Israel Deaconess Hospital, Harvard Medical School, Boston, MA 02115, USA

## Abstract

**Objective:**

To examine biochemical differences in the anterior cingulate cortex (ACC) and insula during the interictal phase of migraine patients. We hypothesized that there may be differences in levels of excitatory amino acid neurotransmitters and/or their derivatives in migraine group based on their increased sensitivity to pain.

**Methods:**

2D *J*-resolved proton magnetic resonance spectroscopy (^1^H-MRS) data were acquired at 4.0 Tesla (T) from the ACC and insula in 10 migraine patients (7 women, 3 men, age 43 ± 11 years) and 8 age gender matched controls (7 women, 3 men, age 41 ± 9 years).

**Results:**

Standard statistical analyses including analysis of variance (ANOVA) showed no significant metabolite differences between the two subject cohorts in the ACC nor the insula. However, linear discriminant analysis (LDA) introduced a clear separation between subject cohorts based on N-acetyl aspartylglutamate (NAAG) and glutamine (Gln) in the ACC and insula.

**Conclusion:**

These results are consistent with glutamatergic abnormalities in the ACC and insula in migraine patients during their interictal period compared to healthy controls. An alteration in excitatory amino acid neurotransmitters and their derivatives may be a contributing factor for migraineurs for a decrease in sensitivity for migraine or a consequence of the chronic migraine state. Such findings, if extrapolated to other regions of the brain would offer new opportunities to modulate central system as interictal or preemptive medications in these patients.

## Introduction

Migraine is a neurobiologic disorder that affects about 27 million women and 10 million men in the US [1]. Migraine attacks manifest themselves from childhood (usually 8–12 yrs.) to old age, with a decline among women during the postmenopausal years. Migraine is a unilateral throbbing headache that lasts 4–72 hours; it is idiopathic, episodic and recurrent [2]. Although the causes of migraine are unknown, it is generally thought that the pain originates from chemical activation of sensory nerves that supply intracranial blood vessels and the meninges [3]. However, the long-term consequences of repeated intermittent attacks of acute migraine on brain function, whatever the origin of the syndrome is, are not well defined.

Two major unanswered questions in the field of migraine relate to (1) *Is there an underlying basis for the increased sensitivity to various stimuli of the migraine brain during *[4,5]*and even between *[6-8]*acute attacks*? and (2) *What is the underlying basis for the recent evidence suggesting that migraine, may predispose to significant functional *[9,10]*and structural changes *[11-15]*in the brain*? One mechanism by which both of these changes may take place is through alterations in neurochemical systems in the brain that are augmented by the repeated acute attacks. Such changes may eventually drive the process on the evolution from acute migraine to chronic daily headaches [16] and also the resistance to drug therapy in the chronic daily headache group [17]. By using magnetic resonance spectroscopy, chemical changes in the brain can be measured in patients. Here we have begun to explore this issue by trying to define these changes during the interictal period in acute intermittent migraine patients for reasons discussed below. A definition of such chemical changes would provide a target for potential interictal therapies that may decrease the severity and/or frequency of migraine and provide a basis for evaluating changes that may take place in the transition to chronic migraine.

A number of recent reports suggest alterations in the interictal migraine brain based on changes in cerebral blood flow [18-20] as well as changes in interictal cognitive function in migraineurs with aura [21]. A wealth of evidence, including measurements demonstrating changes in physiological (i.e., evoked potentials) measures [22-24]), strongly supports the hypothesis of central neuronal hyperexcitability as playing a key role in the pathogenesis of migraine [25]. One potential mechanism for neuronal excitability includes an abnormality of the pre-synaptic release of excitatory amino acid neurotransmitters. Although increased platelet [26,27] and plasma [27,28] levels of neuroexcitatory amino acids including aspartate (Asp), glutamate (Glu), Gln and glycine (Gly) have been reported in migraine patients compared to healthy control subjects [29], these changes are not always good measures or indicators of changes of synaptic glutamate in the brain. In addition, cerebrospinal fluid (CSF) Gln, Gly and taurine (Tau) concentrations are elevated in migraineurs [30] suggesting glutamatergic systems are likely to be altered in the migraine brain. Indeed, given that glutamate is the main excitatory transmitter in the brain excess or under production of glutamate through injury or disease can have pathophysiological effects. The glutamate hypothesis for migraine has been discussed by Ramadan [31] and reviewed recently by Vikelis and Mitsokostkas [32]. Increased synaptic concentrations of excitatory amino acid neurotransmitters may lead to excessive activity at the N-methyl D-aspartate (NMDA) Glu receptor subtype, which may amplify and reinforce pain transmission in migraine and other types of headache. Indeed, low-affinity NMDA receptor (NMDAr) antagonists, such as memantine, have previously been shown to reduce frequency of migraine and tension-type headaches [33].A neuroimaging method capable of assessing potential glutamatergic imbalances in the migraine brain *in vivo *might provide key insights into the true nature of the neurochemical impairment and to monitor its modulation following pharmacotherapy.

^1^H-MRS is a potential candidate for investigating glutamate systems *in vivo *although its application to migraine is relatively sparse in the literature. Functional ^1^H-MRS studies have focused predominantly on changes in the visual cortex [34-36]. Other ^1^H-MRS studies have evaluated metabolite ratios in cluster headache in the hypothalamus and show that N-acetyl aspartate (NAA) to creatine (Cr) ratio is lower in patients with cluster headache vs. chronic migraine or controls [37]. In addition, single-voxel ^1^H-MRS studies have investigated potential cerebellar metabolite alterations demonstrating significantly decreased choline (Cho) levels in migraine patients compared to healthy controls [38]. A similar study detected decreased cerebellar NAA and Glu concentration and increased myo-inositol (mI) levels in familial hemiplegic migraine patients [39]. More recently, a 3.0 T ^1^H-MRS study reported differences in thalamic metabolite ratios in migraine patients compared to healthy controls [40].

Most of these earlier studies employed conventional ^1^H-MRS methodology at a low static magnetic field strength of 1.5 T and none reported changes in multiple brain regions. For the present study we evaluated brain chemistry using medium field 4.0 T ^1^H-MRS in two regions, the ACC and the insula. In addition, we employed a variant of single-voxel 2D *J*-resolved ^1^H-MRS method in an attempt to further enhance spectral resolution and sensitivity, and to provide access to the quantification of an increased number of metabolites [41-43]. The brain regions were selected as an initial focus on evaluating brain metabolites in migraine patients for a number of reasons. The ACC is involved in a number of behaviors [44-46] and is usually implicated in most pain studies [47,48] including migraine [49] and related cognitive components [50]. With respect to the latter it is considered to be involved in reinforcement history [44] that may be relevant in repeated episodes of migraine. The region has been proposed as a model for understanding components of central sensitization of pain [51]. As for the insula, the region is involved in both pain [52] and emotional processing [53], including the unpleasantness of pain [54]. Given the nature of the regions in sensory and emotional processing, we hypothesized that differences in glutamatergic metabolism would be observed when comparing spectral data from patients and healthy controls.

## Methods

### Patients and Controls

**Figure 1:**
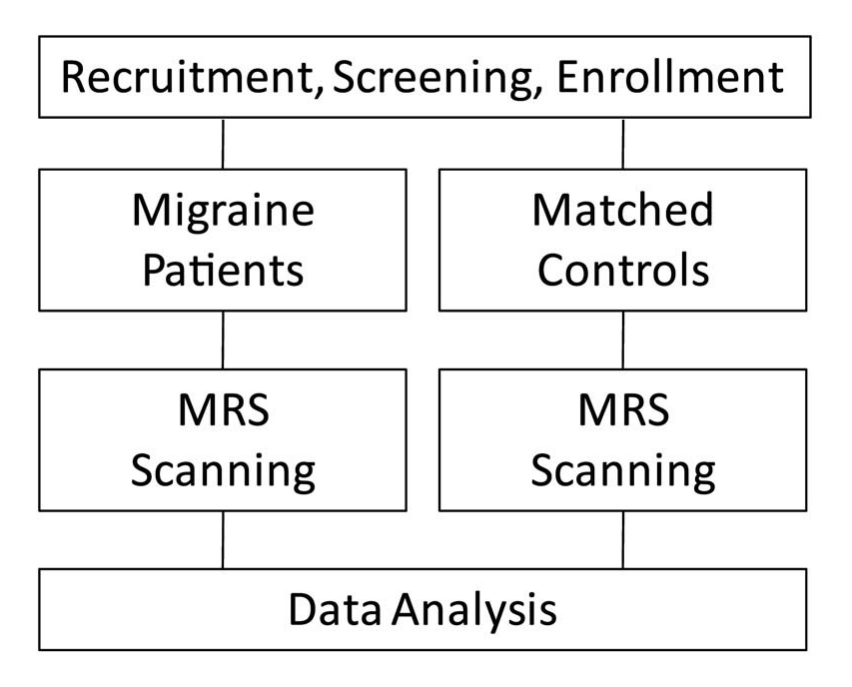
**Patient Enrollment and Scanning**.

The overall experimental approach is shown in Figure 1. The local Institutional Review Board of McLean Hospital approved this study (IRB), which met the criteria for investigations in human subjects based on the Helsinki Accord . Patients presenting with acute episodic migraine (AEM; n = 12) and healthy age (n = 8), gender matched controls (HC) were recruited for the study.

### Data Acquisition

#### MRI Procedures

All measurements were performed at McLean Hospital on a Varian 4.0 T Varian Unity/INOVA whole body MRI scanner (Varian Inc., Palo Alto, CA, USA). A birdcage design radiofrequency (RF) head coil tuned to 170.3 MHz was used for RF transmission and signal reception. Three orthogonal gradient-recalled scout images were initially obtained to ensure optimal head positioning within the coil (TR/TE = 30/10 ms, field-of-view = 24 × 24 cm, matrix = 256 × 128, slice thickness = 5 mm). Manual global shimming subsequently was performed until the unsuppressed water resonance linewidth was ≤ 25 Hz. High-contrast, 3D mpFLASH (magnetization-prepared, fast, low-angle shot) T_1_-weighted axial, coronal and sagital MR images (TE/TR = 6.2/11.4 ms, FOV = 24 × 24 × 8 cm, in-plane resolution = 0.94 × 1.88 mm, slice thickness = 5 mm, readout points = 512, flip angle = 11°) then were acquired to more accurately delineate brain substructures and to enable more accurate positioning of the spectroscopy voxel within the region-of-interest (ROI).

#### MRS Procedures

**Figure 2:**
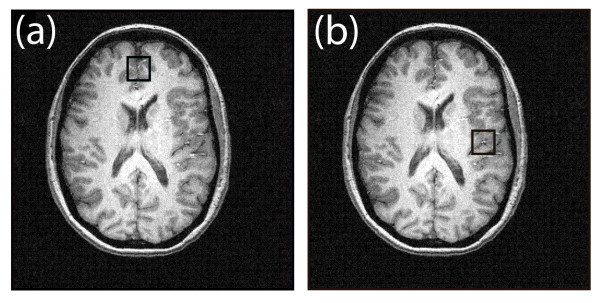
**Voxel Placement for Spectral Analysis**. Axial high-resolution T_1_-weighted FLASH MR images recorded from a 42 year-old male migraine patient showing a 8-cm^3 ^spectroscopy voxel (black box) positioned within (a) the ACC and (b) the left insula.

For ACC ^1^H-MRS measurements, a cubic 8 cm^3 ^voxel was positioned within the predominantly midline gray matter of the ACC (see Figure 2(a)). For insula measurements, a cubic 8 cm^3 ^voxel was positioned contralateral to the side of the subjects headache (see Figure 2(b)). Automated routines initially were used to optimize spatial localization RF pulse flip angles for each ROI, and localized manual shimming subsequently was applied to until the full width at half maximum of the unsuppressed water-water peak was ≤ 12 Hz. Water-water suppression was achieved using a four-pulse WET module [55]; A modified PRESS sequence was used for 2D *J*-resolved ^1^H-MRS acquisitions, which utilized numerically optimized sinc RF waveforms for excitation (duration = 3.0 ms; BW = 2850 Hz) and refocusing (duration = 6.0 ms; BW = 1145 Hz). 2D *J*-resolved ^1^H-MRS spectra were acquired from each ROI using the following acquisition parameters (TR = 2000 ms, TE range = 30 – 260 ms, ΔTE = 10 ms, NEX = 16 per TE, dummy scans = 4, 1024 complex points). Data processing was performed offline with each of the 24 sub-TEs being stored separately for preprocessing purposes. The total measurement time for each 4.0 T study including MRI, shimming and ^1^H-MRS procedures was approximately one hour.

### Data Processing

#### Spectral Analyses

The 2D *J*-resolved time-domain data were transferred to a personal computer and preliminary data pre-processing and formatting steps performed using home-written code written in C programming language. The processing specifics were as follows. A raw 2D data matrix was constructed and zero-filled to yield a 2048 (F2) × 64 (F1) matrix and a fast Fourier transformation subsequently was applied to the 2048 column vectors along the F1 dimension. The 64 row vectors then were extracted from the resulting interferrogram and each row was converted to Linear Combination Model (LCModel) format for spectral fitting and measurement of metabolite peak integrals [56]; version 6.0-1). For spectral fitting with LCModel, we utilized GAMMA-simulated [57] theoretical basis sets for alanine (Ala), aspartate (Asp), choline (Cho), creatine (Cr), N-acetyl-aspartate (NAA), N-acetyl-aspartyl-glutamate (NAAG), γ-aminobutyric-acid (GABA),, glutamine (Gln), glutamate (Glu), glutathione (GSH), glycerophosphocholine (GPC), glycine (Gly), lactate (Lac), myo-inositol (mI), phosphocholine (PC), phosphocreatine (PCr), scyllo-inositol (sI), serine (Ser), and taurine (Tau). We used GAMMA to generate 24 theoretical, TE-stepped spectra ranging from 30 ms to 260 ms in 10 ms increments, and each GAMMA spectrum was modeled with a 2 kHz spectral bandwidth, 1024 complex pairs and a 2 Hz Lorentzian lineshape. Each of the 24 spectra were zero and first-order phased with no baseline roll. A formate peak at 8.45 ppm and a 3-(trimethylsilyl)-1-propane-sulfonic acid (TSPS) reference peak at 0.0 ppm were also modeled in order to mimic the standard LCModel stock solution required for basis-set generation [58]. For each GAMMA-simulated metabolite TE-series, we zero-filled each complex time-point in each FID out to 128 TE points and apodized with an exponential filter to approximate the metabolite T_2_-decay at 4.0 T. These TE-specific metabolite FIDs were converted into separate LCModel basis sets for each metabolite thus providing a means to quantify the whole 2D surface of the in vivo datasets [59]. The LCModel/GAMMA-derived 2D surface metabolite integrals were normalized to the total Cr integral. 2D *J*-resolved ^1^H-MRS-derived ACC metabolite:Cr ratio reliability indices were taken from a previous study [59], which employed the identical sequence and similar parameters to that described for the present study. In brief, ten healthy adult subjects (5 males and 5 females; ages 18–35) were scanned three times in a one week period, except for two females whose third scan was 4 weeks later at the same phase of the menstrual cycle. Within-subject coefficients of variation (CV) were calculated as the standard deviation/mean of the three scans.

#### Image and Voxel Segmentation

All image analyses were performed using the freely-available FMRIB Software Library (FSL; for an overview see [60]). Tissue segmentation of the 3D T_1_-weighted FLASH MR images into grey matter (GM), white matter (WM) and cerebrospinal fluid (CSF) used FSL's fast automated segmentation tool (FAST; [61]). The ACC and insula 8-cm^3 ^voxels subsequently were extracted from segmented images using the FSL 'fslroi' tool, and voxel GM, WM and CSF fractions were determined from the generated image histogram.

#### Assessment of motion effects during 1H MRS

The duration of each TE-averaged ^1^H-MRS acquisition was 13 minutes for each brain region and subject/head motion throughout the measurement was a potential confound between datasets and subject cohorts. Motion effects would lead to modification of the local B_0 _field over the ROI potentially leading to modulation of peak linewidth throughout the ^1^H MRS scan. To evaluate motion effects we measured the NAA 2.01 ppm methyl proton signal linewidth for each of the 24 TE steps across all subjects for both brain regions. The intrasubject mean NAA peak linewidth then was calculated for both the ACC and insula and the group mean FWHM values were statistically compared (unpaired t-test). In addition, the intrasubject NAA peak linewidth CV was calculated using all 24 TE steps for both brain regions, and the group mean CV values were evaluated (unpaired t-test).

#### Statistical Analyses

Standard statistical analyses including ANOVA were performed using Origin version 7.5 (OriginLab Corp., Northampton, MA, USA). In addition LDA was used to evaluate separability of the two subject cohorts (migraine and control) using metabolite measurements across the two groups in each ROI. This method enables multivariate extraction of differences between groups. Home-written scripts were used for performing LDA analyses (MATLAB, The Mathworks, Natick, MA, USA). For each ROI a set of two metabolites were selected that had the best group discrimination ability via a stepwise forward search using the hotelling T2 statistic. We restricted our search to only two responses so as to enable robust estimation of the LDA covariance.

## Results

### Patients and Controls

As noted in Table 1, patients recruited all had migraine and were compared with age-gender matched controls. Of the 12 patients recruited, 1 dataset could not be used owing to poor spectral quality. A second patient had also been taking methylene sulfonyl methane (MSM) supplements and a large-amplitude MSM-specific resonance was observed at 3.1 ppm. Data from that subject was also excluded from the final analyses.

**Table 1:** Epidemiology of Patients

**Age**	**Gender**	**Migraine Frequency (per month)**	**Migraine History (years)**	**Migraine Prophylaxis**
30	F	3	4	rizatriptan, ibuprofen aspirin/butalbital/caffeine acetominophen

49	F	2 – 3	28	eletriptan, naproxen aspirin/butalbital/caffeine topiramate

40	F	4	7	muscle relaxants

57	M	2 – 7	50	ibuprofen, amitriptyline caffeine/ergotamine fexofenadine

32	F	2 – 3	6	acetominophen

42	M	2 – 3		elitriptan, verapamil

58	F	2 – 3	45	naratriptan

51	M	3	45	sumatriptan, eletriptan acetominophen/aspirin/caffeine hydrocodone/ibupfrofen

28	F	4 – 8	12	indomethacin, naratriptan sumatriptan, eletriptan

47	F	2	10	omeprazole

### MRI/MRS

Figure 2 shows the high-resolution axial MR images recorded from a 32 year-old female migraine patient and displays positioning of the spectroscopy voxel within the (a) ACC and (b) left insula. The percentage (mean ± SD) of GM in ACC and insula was 69 ± 8% and 56 ± 5% (HC), and 72 ± 6% and 59 ± 7% (AEM), respectively. The percentage (mean ± SD) of GM in ACC and insula was 69 ± 8% and 56 ± 5% (HC), and 72 ± 6% and 59 ± 7% (AEM), respectively. Similarly, the percentage (mean ± SD) of WM in ACC and insula was 16 ± 6% and 37 ± 5% (HC), and 15 ± 6% and 33 ± 5% (AEM), respectively. For both brain regions, standard ANOVA showed that at the *P *< 0.05 level no significant differences in tissue content existed between the two groups. However, the WM fraction was significantly higher in the insula voxel compared to corresponding ACC voxel for both subject cohorts (ANOVA: *P *< 0.01).

**Figure 3:**
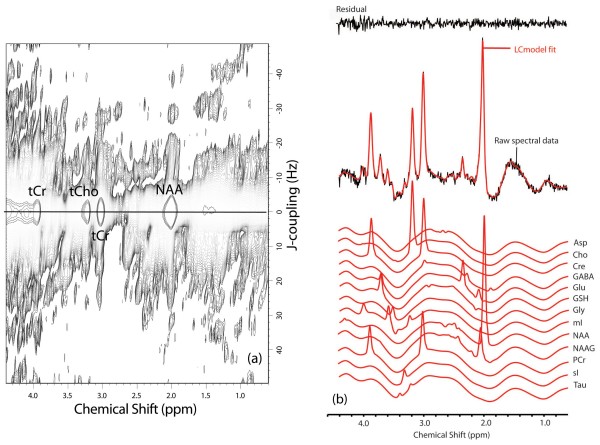
**(a) Left Panel: 2D *J*-resolved ^1^H-MR spectrum recorded from the voxel shown in figure 2b**. Only the large singlet resonances are labeled at F1 = 0 Hz although information from *J*-coupled metabolites is spread over the 2D surface. The 2D spectrum is presented in magnitude mode for presentation purposes, and is characterized by two orthogonal frequency axes: chemical shift (F_2_) and *J*-coupling (F_1_) dimensions. Exponential apodization was applied along F2 (line broadening = 3 Hz) whereas a sinebell-squared apodization filter (30° phase-shifted) was applied along the F1 axis. The chemical shift axis has been expanded to show the 0.6 – 4.4 ppm region whereas the full *J*-coupling dimension (± 50 Hz) is presented. The dominating singlet resonances corresponding to the NAA, total Cr and total Cho methyl groups are labeled and reside perpendicular to F_1 _= 0 Hz (solid black line) at 2.0, 3.0 and 3.2 ppm, respectively. Lipid signals that probably arise due to chemical shift displacement are identified at and around 1.4 ppm (F_1 _= 0 Hz) and the total Cr methylene resonance is observed at 3.9 ppm. (b) Right Panel: LCModel analysis of a single row extracted from F_1 _= 0 Hz. The raw data, LCModel fit, residual and individual metabolite fits are displayed. It is important to note that the 1D ^1^H-MR spectrum is real data, which is the data type required for LCModel analysis. The LCModel fit (solid red line) for the F_1 _= 0 Hz extraction is overlaid on the raw data and the residual (LCModel fit minus raw data) is displayed at the top of the figure. The residual is free from large subtraction artifacts and clearly illustrates the high-quality spectral fit achieved using the described quantification methods. Data extracted from the center of the F_1 _dimension in this manner is entirely equivalent to signal averaging all 24 TE steps, a procedure often referred to as TE-averaging. TE-averaged ^1^H-MRS spectra typically show the large singlet methyl (CH_3_) resonances of NAA, total Cr and total Cho with significant attenuation of resonances *J*-coupled metabolite species. (see text for more details).

For the control group, the FWHMs (mean ± SD) measured for the unsuppressed water resonance were 9.75 ± 1.6 and 9.25 ± 1.0 Hz for the ACC and insula voxels, respectively. For the migraine group, the FWHMs (mean ± SD) measured for the unsuppressed water resonance were 10.2 ± 2.0 and 10.0 ± 1.1 Hz for the ACC and insula voxels, respectively. Figure 3(a) shows a 2D *J*-resolved ^1^H-MRS representation of data acquired from the left insula in the same subject. Figure 3(b) then shows a ^1^H-MR spectrum (labeled 'Raw spectral data'), which was produced by extracting a single row from the 2D *J*-resolved spectrum at F_1 _= 0 HzThe scaled LCModel fits for thirteen individual metabolites are displayed at the bottom of Figure 3(b) and each metabolite fit is superimposed on a baseline calculated by the LCModel software. Lipid contamination is once again observed at and around 1.4 ppm although the broad peak has been treated as a macromolecular resonance and fitted by LCModel. The 1D and 2D ^1^H-MRS data presented in Figure 3 is representative of the spectra acquired from all brain regions in both subject cohorts. The above information indicates high spectral quality and therefore a basis for robust quantification via LCModel.

### Assessment of Motion Effects during 1H MRS

Table 2 shows the group averaged NAA peak linewidth and CV data for the ACC and insula MRS voxels in the control and migraine patient populations. Note that the group mean FWHM values appear higher than those provided earlier for the unsuppressed water resonance, an observation that is likely due to signal contribution from other metabolite resonances including Gln, Glu, GABA and NAAG to the NAA 2.01 ppm resonance, which increases the 'raw' NAA signal linewidth particularly for the lower TE values. For each subject, the mean NAA peak FWHM and linewidth CV calculations were computed using all 24 TE steps (see methods section for more details). It is clear from Table 2 that no statistically significant differences in NAA signal FWHM or its CV were detected for both brain regions between the patient populations, inferring that any existing motion effects were comparable between groups.

**Table 2:** Assessment of NAA peak linewidth

**Subject Cohort**	**Brain Region**	**FWHM (Hz; Group mean ± SD)**	**CV (%; Group mean ± SD)**
Migraine	ACC	18 ± 5.1^†^	19.4 ± 4.6^††^

Control	ACC	18 ± 3.7^†^	19.1 ± 5.2^††^

Migraine	Insula	15.3 ± 8.3*	29.3 ± 14.1**

Control	Insula	17.9 ± 3.7*	34.5 ± 12.4**

(see text for details; ^†^P = 1; ^††^P = 0.91; *P = 0.51; **P = 0.36)

### Statistical Analyses

**Figure 4:**
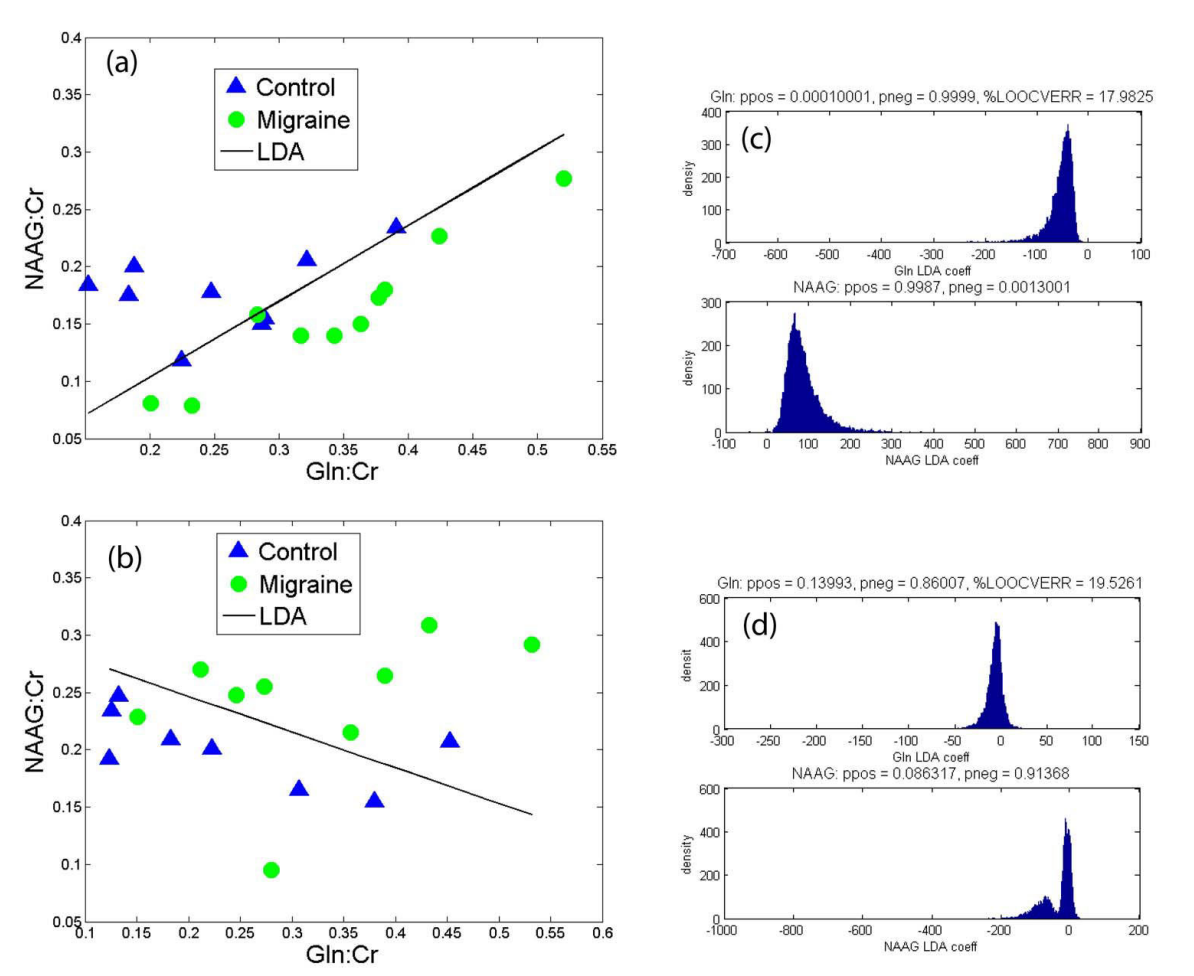
**Linear Discriminant Analysis (LDA)**. (a) The figure depicts 2 metabolite ratios (e.g., NAAG/Cr and Glu/Cr) that separate controls from migraineurs through the black line in the cingulate. (b) Similar data is shown for the insula. (c) and (d) show a bootstrap calculation for assessment of statistical accuracy of the estimated LDA coefficients for the ACC and insula, respectively.

Standard statistical analyses showed no significant metabolite differences between the two subject cohorts. However, by using LDA, we show that we can separate patients vs. controls as shown for the ACC and insula in figures 4(a) and 4(b), respectively The classification is achieved using the metabolites NAAG and Gln for both brain regions, and the within-subject CV's for these particular metabolites were 23 and 21%, respectively. For each ROI, we also assessed the statistical accuracy of the estimated LDA coefficients using a bootstrap calculation [62]. The sampling unit for bootstrap included the set of metabolite measurements and a group label (migraine/control) sampled with replacement from the original data. We fitted the LDA model to each bootstrap data set and calculated a distribution of the LDA coefficient value for each metabolite. This distribution was then used to estimate the probability of LDA coefficient values being positive or negative. Small values of the coefficient being positive indicate a statistically significant negative coefficient and vice versa. Figures 4(c) and 4(d) show the bootstrap calculations for the ACC and insula, respectively. Since LDA allows for the simultaneous comparison of observables the approach allows us to utilize brain metabolite ratio's to establish differences between cohorts i.e., migraine and healthy controls.

## Discussion

Here we report novel differences in ^1^H-MRS defined levels of metabolites in the ACC and insula measured in the interictal period of migraine patients. Although conventional descriptive statistics yielded no differences on the analysis of the spectra, a LDA demonstrated significant differences between migraine subjects and age-gender matched controls. This type of analysis allows for the determination of discrimination of two or more groups (e.g., migraine vs. healthy) based on Cr-normalized levels of specific metabolites. This analysis separated out a relationship between NAAG and Gln within the ACC and insula during their interictal period. NAAG is the most abundant peptide neurotransmitter in the mammalian CNS [63] being synthesized exclusively in neurons from NAA and Glu by NAAG synthetase. In addition to its role as a neurotransmitter, NAAG is a source of Glu [64] and like NAA is thought to play a role as a major osmolyte in the vertebrate brain [65,66]. Glutamine on the other hand is synthesized exclusively in glial cells from Glu and ammonia by the enzyme glutamine synthetase. Subsequently, Gln is released back into the extracellular space, shuttled back into neurons and converted to Glu by glutaminase. The Glu that is regenerated may then go on to play a direct role in excitatory neurotransmission, packed and stored in vesicles or incorporated into NAAG. An intriguing observation in the present study is the LDA-detected classification of migraine patients and control subjects for two different brain regions based on NAAG and Gln, which are closely linked by this excitatory neurotransmitter system. Interestingly, the ACC and insula LDA plots show oppositely signed gradients, an observation that might be explained by (i) the significant tissue type differences within ACC and insula voxels detected by image segmentation and (ii) the known uneven distribution of Gln and NAAG throughout the brain and within brain tissue type [67]. The measured changes in these excitatory amino acid neurotransmitters (NAAG) and related species (Gln) provide some insights into altered central nervous system (CNS) mechanisms in migraine and may contribute to abnormal CNS processing including changes during the migraine state (e.g., process of central sensitization [68,69], progressing from acute episodic to chronic/daily migraine [70] or abnormalities during the interictal period [71-74]. We did not detect direct differences in Glu levels between controls and migraine patients although preferential storage of excess synaptic Glu in the form of Gln and/or NAAG might explain comparable Glu levels within the two cohorts. Note that a previous ^1^H-MRS study showed decreased cerebellar Glu levels in migraine patients compared to healthy controls [39] yet similar cortical ^1^H-MRS findings have not been reported to date.

A growing body of preclinical and clinical data supports the notion of aminergic dysfunction in migraine headache including alterations in both the glutamatergic and glutaminergic systems [31,32]. For example, NMDA receptor antagonists inhibit cortical spreading depression in the rat brain [75]. Cerebrospinal fluid [76] and plasma [27] Glu and Gln levels are increased in chronic migraine patients, although no such data is available for episodic migraine (i.e. our population). It has been postulated that increased brain Glu leads to cortical hyperexcitability typical of migraine [77,78] and potential pharmacological targets for migraine therapy include the ionotropic (NMDA, AMPA and kainate) and metabotropic glutamate receptor antagonists [79]. The use of a tridimensional personality questionnaire in migraine and tension-type headache clinical sub-populations has shown that glutaminergic dysfunction might also be a specific feature associated with migraine headache [80]. The development of novel pharmaceutics that can modulate the glutaminergic system and block central and peripheral sensitization might be efficacious for treating migraine. It is also worth noting that, although little is known in the literature for a potential role of NAAG in migraine, there may be a potential role for NAAG antagonists (via mGluR3 receptor blockade) for the therapy of migraine.

A number of reports indicate that modulation of the glutamatergic system in the ACC takes place following pharmacological or sensory manipulation. Alterations in ACC neurons may be dependent on prior events that change or modulate neuronal activity. For example, drugs may decrease levels of glutamate in the ACC [81] and excitatory synapses into the ACC are in part NMDA mediated changes in this region [82]. In addition, amputation of a hind paw digit in rats results in a loss of activity-dependent long-term depression in the ACC [83] and potentiation of sensory responses [84]. NMDA receptors in the ACC mediates pain-related aversion [85]. Thus, in migraine patients either as a result of intermittent pain or medications, ACC glutamatergic impairment would account for an increase in activation in this region. In data from another report we observe increased sensitivity in the descending modulatory systems in the brainstem in interictal migraine patients vs. controls [86]. In functional imaging studies of pain, activation in the insula is observed and it has been suggested that the region has important contributions to both pain and emotional processing [87,88]. However, ^1^H-MRS detected changes in this region in the interictal period have not been reported.

For the present study, we chose to use a 3D localized variant of *J*-resolved ^1^H-MRS, a method that has been shown to enhance spectral resolution at several field strengths including 1.5 T [42,43], 3.0 T [89] and 4.0 T [90]. Increased spectral resolution is achieved as *J*-coupled metabolite resonances are effectively spread over a 2D surface whereas uncoupled peaks remain along F_1 _= 0 Hz. Glutamine contains a single methine (CH; 3.75 ppm) and two methylene (CH_2_; 2.1 and 2.4 ppm) groups and each proton resonance is split owing to *J*-coupling effects [91]. It follows that, for 2D *J*-resolved ^1^H-MRS data, glutamine shows multiple proton resonances across the 2D surface. In combination with LCModel fitting and GAMMA-simulated basis sets, we use information from the whole 2D datasets and this approach further improves multiple-metabolite quantification of 2D ^1^H-MRS data. Recently we applied these methods *in vivo *and demonstrated their utility for reliably measuring brain glutamate and glutamine levels [59]. NAAG, a dipeptide composed of NAA and Glu joined by a peptide bond, also benefits from the 2D ^1^H-MRS approach. The major resonance of NAAG is its CH_3 _resonance at 2.04 ppm that appears as a shoulder on the dominating NAA CH_3 _2.0 ppm peak. In conventional ^1^H-MR spectra, this chemical shift region is further complicated by underlying *J*-coupled resonances of Gln, Glu and GABA, and a major advantage of 2D *J*-resolved data is the fact these *J-*coupled metabolite resonances are shifted away from the F_1 _= 0 Hz axis. This yields a cleaner chemical shift region that is essentially comprised of NAA and NAAG CH_3 _singlet peaks, both of which are more reliably fitted by the described LCmodel template and fitting procedures.

It is certainly true that MRS studies are limited by the relatively low SNR of the spectra and some studies of chronic pain patients have noted larger between group differences. However, the methods that were employed in the present study were designed to allow the detection and quantitation of a larger number of lower concentration metabolites. This has to make observations that would not have been possible using more standard methods. In addition the interictal migraine group may differ from the chronic pain state in that it produces prolonged and continuous brain changes that manifest in profound structural [15] and functional changes [92,93].

## Conclusion

Our results have examined changes in biochemical concentrations in two brain regions in which we report some abnormal relation between a pair of peaks in only two brain regions. Nevertheless, the approach may be used for determining an underlying alteration in the biochemical dysfunction in this group of migraine patients would allow for specific therapeutic interventions that may normalize these changes in the interictal period rather than current approaches of treating migraine during the ictal period [94,95].

## Competing interests

The authors declare that they have no competing interests.

## Authors' contributions

AP implemented MRS acquisition and data analysis methods, participated in organization of study design, carried out MRS acquisitions and drafted most parts of the manuscript. LB helped with overall study design and oversaw data analysis procedures. GP performed statistical data analyses. ST was entirely responsible for subject screening and enrollment. EJ provided and oversaw 2D MRS data analysis methods. RH was involved in the overall study design and oversaw data analysis procedures. PR was helped with the original study design and oversaw MRS data acquisition and analysis procedures. RB contributed to direction of the study and oversaw data analysis and interpretation. DB contributed to conceptual framework and overall direction of the study including overseeing data analysis, interpretation and manuscript preparation. All authors have read and approved the final manuscript.
